# Associated Risk Factors and Diagnostic Value of Fiberoptic Bronchoscopy for Protracted Bacterial Bronchitis in Children

**DOI:** 10.1155/2023/8116651

**Published:** 2023-07-05

**Authors:** Rong Zhang, Li Wang, Chen Gong, Hui Gao, Wenhong Li, Chenrong Bian, Jiaying Zhao, Shenggang Ding, Yulin Zhu

**Affiliations:** Department of Pediatrics, The First Affiliated Hospital of Anhui Medical University, Anhui 230000, China

## Abstract

**Objective:**

Untreated protracted bacterial bronchitis (PBB), a chronic wet cough prevalent in children, may lead to chronic suppurative lung disease. However, clinical diagnostic criteria are currently nonspecific; thus, PBB may be misdiagnosed. Thus, we assessed the diagnostic value of fiberoptic bronchoscopy (FOB) and the risk factors associated with PBB.

**Methods:**

Children with chronic cough at The First Affiliated Hospital of Anhui Medical University from January 2015 to May 2020 were enrolled and allocated to a suspected PBB (*n* = 141) or a non-PBB (*n* = 206) group. All children underwent extensive laboratory, chest imaging, and allergen tests. Children with suspected PBB underwent FOB with bronchoalveolar lavage; lavage and sputum samples were cultured.

**Results:**

All 347 children had a chronic wet cough for approximately 2 months. Of 141 children with suspected PBB, 140 received FOB with bronchoalveolar lavage. Visible tracheal changes included pale mucosa, mucosal congestion, edema, swelling, and increased secretions attached to the wall. Sputum was visible primarily in the left main bronchus (78.7%), left lower lobe (59.6%), right upper lobe (62.4%), and right lower lobe (64.5%). Sputum properties and amounts significantly differed between children with vs. without PBB (*P* < 0.05). Dermatophagoides (odds ratio (OR), 2.642; 95% CI, 1.283–5.369), milk protein (OR, 2.452; 95% CI, 1.243–4.836) allergies, and eczema (OR, 1.763; 95% CI, 1.011–3.075) were risk factors significantly associated with PBB.

**Conclusion:**

Dermatophagoides, milk protein, and eczema were associated with an increased risk of PBB. Sputum distribution and tracheal wall changes observed through FOB may distinguish PBB and assist in its diagnosis.

## 1. Introduction

Of all childhood illnesses, coughing is the most common one [1]. Usually, coughing for more than 4 weeks is called chronic cough [2]. For chronic cough, prevalence is typically >10% across most surveys, ranging from 7% to 33% [3]. Yu et al. reported that even healthy children have about 10 times coughs a day, and it occurs most often in the daytime [4]. There is evidence that allergens may be cough-related factors [5]. Approximately, 10%–22% of preschoolers develop a chronic cough in the absence of a cold [4]. Cough variant asthma, upper airway cough syndrome, and protracted bacterial bronchitis (PBB) belong to the category of chronic cough [6], but the clinical manifestations of PBB are not specific. PBB is the most easily missed diagnosis of chronic cough in children owing to insufficient understanding of this condition [7]. Thus, in the clinical differentiation of chronic cough etiology, it is important to be aware of this lung condition so that appropriate therapy may be started before associated complications arise. Early detection and treatment are critical because PBB may be a precursor to chronic suppurative lung disease when the disease repeatedly irritates the airway.

The concept of PBB was first proposed by Verhagen and de Groot as a chronic endobronchial infection caused by bacteria [8]. The clinical manifestations are wet cough, especially paroxysmal cough, which may spit out sputum, accompanied by wheezing and a runny nose during the day and after exercise [9]. However, these are general clinical manifestations that can also be seen in other chronic cough diseases, such as asthma and upper airway cough syndrome [10]. The pathogenic bacteria causing PBB are mainly common respiratory pathogens, including undifferentiated *Haemophilus influenzae* and *Streptococcus pneumoniae* [10]. The diagnostic accuracies of PBB and other chronic cough diseases need to be improved. Many pediatricians are not familiar with PBB [10]. The clinical diagnostic criteria for PBB are not typical of the disease, which makes it possible to distinguish it from other diseases that cause chronic cough [12] and may cause some cases with atypical manifestations to be missed [10].

Although the current definition of PBB has increased our understanding of chronic wet cough in children, there are still many problems that have not been effectively solved [11]. Fiberoptic bronchoscopy (FOB) may help to better define PBB. FOB is currently an integral part of the management of various lung and airway diseases in children and can achieve the purpose of diagnosis or treatment [12]. It can directly observe changes in the bronchial mucosa and collect samples of secretions from the distal airway by bronchoalveolar lavage (BAL) for pathological and microbiological examination [13]. If the clinical diagnosis of PBB is based on general presentation, some children with postinfection cough or non-PBB would be misdiagnosed as having PBB and would likely be treated with antibiotics, leading to the abuse of antibiotics. However, if the diagnostic criteria for PBB are strictly followed, a diagnosis of PBB may be missed or treatment delayed, affecting the prognosis due to the length of time needed for laboratory testing and specimen transport. Some clinicians argue that the diagnostic criteria for PBB are poorly defined and others call for further research [14]. Thus, this study aimed to provide extensive laboratory data, including the results of bacterial cultures of bronchoalveolar lavage fluid (BALF) and other FOB findings to offer a detailed description of the clinical manifestations, laboratory characteristics, and typical microscopic manifestations of PBB to aid in the clinical diagnosis of it.

## 2. Materials and Methods

### 2.1. Participants

A retrospective analysis was conducted on a total of 347 children (228 males and 119 females; median age of 3.9 years) with chronic cough recruited from The First Affiliated Hospital of Anhui Medical University between January 2015 and May 2020. Most of the children had received various courses of antibiotics prior to hospital admission, but none had a congenital pulmonary abnormality. Written informed consent was obtained from all parents or guardians of all children who underwent the FOB examination.

### 2.2. Inclusion Criteria and Exclusion Criteria

Previous diagnoses of PBB include the following: (a) a wet cough lasts longer than 4 weeks, (b) a BALF culture results of identifiable lower airway bacterial infection, (c) the cough improved after 2 weeks of antibiotic treatment, and (d) absence of an alternative specific etiology [8] (Table 1). The diagnostic criteria for pathogenic microorganisms of PBB are as follows: (1) a wet (sputum) cough lasting >4 weeks, (2) evidence of lower respiratory tract infection, (3) positive bacterial culture of sputum or BALF, with a colony count ≥10^4^ CFU/mL, and (4) the cough improved after 2 weeks of antibiotic treatment [15]. The following additional definitions are used in clinical practice: PBB-extended is PBB-micro or PBB-clinical (Table 1) requiring 4 weeks of antibiotic treatment for cough resolution, and recurrent PBB is defined as >3 episodes of PBB per year [15].

#### 2.2.1. Inclusion Criteria

All the children included in this study met the following conditions: (1) children with chronic cough hospitalized in the Department of Pediatric Respiratory Hematology and Endocrinology of The First Affiliated Hospital of Anhui Medical University from January 2015 to May 2020; (2) chronic wet cough for more than four weeks. The included children were divided into two groups, and the PBB group satisfied the following: (1) signs of lower airway infection confirmed after fiberoptic bronchoscopy with alveolar lavage and (2) response to two weeks of antibiotic (amoxicillin-clavulanate) therapy; cough resolved to different degrees than before. Children with chronic wet cough excluding the PBB group were included in the non-PBB group (*n* = 206), which included cough-variant asthma (*n* = 39) and upper airway cough syndrome (*n* = 78).

#### 2.2.2. Exclusion Criteria

(1) Patients with systemic diseases, such as malignant blood system diseases and perennial bedridden due to spinal muscular atrophy; (2) patients with immune deficiency disease, genetic metabolic disease, and congenital heart disease; (3) severe failure of organ function (such as liver and kidney functions); and (4) parents refuse to improve relevant examination and refuse to accept follow-up.

### 2.3. Routine Examination

Before the FOB examination, several routine examinations will be performed on the child including the following aspects: (1) blood routine detection, coagulation function detection, C-reactive protein, and procalcitonin level; (2) chest radiograph, CT, ECG, and lung function examination; (3) 11 tests of immune function; (4) tests for reactions to common environmental allergens, including dust mites, pollen, milk protein, and seafood; and (5) eczema history, supplementary food, and parity.

### 2.4. Prehospital Treatments

Despite the use of antibiotics, steroids, budesonide suspension aerosol inhalation, and other treatments, the respiratory symptoms of the child were not effectively controlled.

### 2.5. Fiberoptic Bronchoscopy

All patients received budesonide suspension and albuterol suspension inhalation before surgery. Before fiberoptic bronchoscopy with alveolar lavage, all children were given intravenous atropine to reduce the secretion of respiratory mucosa mucus. Fiberoptic bronchoscopy with alveolar lavage was performed under local anesthesia, and the duration of the examination was generally controlled at about 30 minutes. Local anesthesia involves spraying lidocaine solution into the nasal cavity and throat through a watering can, spraying into the main bronchus and left and right bronchus via a fiberoptic bronchoscope, and an intravenous infusion of midazolam (0.1–0.3 mg/kg) for sedation [16]. Before surgery, the operator will determine whether there are signs of large sheet shadow change in the lungs according to the chest image of the child, so that the relevant lesion area can be repeatedly lavage during the operation. For children over 5 years old, use FB-15V bronchoscopy (with inner diameter 2.0 mm and outside diameter 4.8 mm), for children under the age of five years old, use specification FB-10V bronchoscope (with inner diameter 1.2 mm and outside diameter 3.6 mm) for fiberoptic bronchoscopy with alveolar lavage. The child was placed in a supine position on the examination table before surgery, after a lidocaine spray to the nasal cavity; from the right nostrils successively into the nasal cavity, you can intuitively see the general situation of the nasal cavity, if there is no turbinate hypertrophy and whether the nasal mucosa is inflamed. Then, it enters the glottis and trachea in turn, generally entering the right main bronchus and the right subsegment of each bronchus, and then entering the left main bronchus and the left subsegment of each bronchus. During the operation, normal saline (0.5 mL/kg, total dose < 5 ml) was injected into the left and right main bronchus and each subsegment bronchus, repeated lavage was performed, and BALF were collected. In addition to the location of the lesion indicated by the chest tablet or chest CT, the operator also chose the area with the most abundant and obvious secretions for repeated lavage. After the examination, BALF samples are generally sent to the microbiology laboratory and emergency laboratory of the hospital within 2 hours for BALF-related tests. After surgery, the attending physician will evaluate the clinical manifestations of the children before and after fiberoptic bronchoscope with alveolar lavage and evaluate whether there is a need for a pulmonary imaging review based on remission according to the clinical manifestations.

### 2.6. Follow-Up Visits

Telephone follow-up visits were conducted 1, 6, and 12 months after hospital discharge to request information on medication adherence and cough resolution time of children to determine the effectiveness of antibiotic therapy.

### 2.7. Statistical Analysis

SPSS 23.0 statistical software was used for data analysis. Comparisons between two groups were performed using independent samples *t*-tests, data with a nonnormal distribution are represented by medians and interquartile ranges (Mann–Whitney test), and counted data are denoted as percentages. Potential PBB risk factors were analyzed by chi-square tests and logistic regression. *P* < 0.05 was considered statistically significant.

## 3. Results

### 3.1. General Clinical Features

The median age of the included children with PBB was 3.90 (1.75, 6.03) years, and the male to female ratio was approximately 3 : 2. No statistical differences were observed between the age or gender groups (*P* > 0.05). Although parents typically reported wheezing, pulmonary auscultation wheezing sounds are rare and more common are “chest whoosh” and sputum sounds in the throat. It was also commonly reported that the increase in sputum led to faster and “thicker” breathing when the child was lying down. In our study, about 48.9% of children with PBB had wheezing due to lung auscultation by clinicians.

The duration of the cough that had been present prior to this study did not differ substantially between children with PBB (*n* = 141) vs. those without PBB (*n* = 206). There was no difference in the method of feeding (i.e., breast, formula, or mixed) between children with vs. without PBB. Although the probability of bronchomalacia may be markedly increased among children who are not firstborn due to trace element and a lack of calcium supplements [8], there was no significant difference between children with PBB vs. non-PBB, whether or not they were firstborn (Table 2).

### 3.2. Associated Risk Factors

Children with a history of allergies received 23 allergen tests. Dermatophagoides allergy was substantially higher in children with PBB than in children without PBB (*P* < 0.05). The proportion of children with PBB having a milk protein allergy (19.1%) was markedly higher than that for children without PBB (7.8%). Eczema was also significantly different between the two groups: 24.8% of children with PBB vs. 15% of children without PBB. Logistic regression analyses indicated that milk protein allergy (*P*=0.01), eczema (*P*=0.046), and dermatophagoides allergy (*P*=0.008) were statistically significantly different in children with PBB vs. children without PBB (Table 3).

### 3.3. FOB and BAL

**Figure 1:**
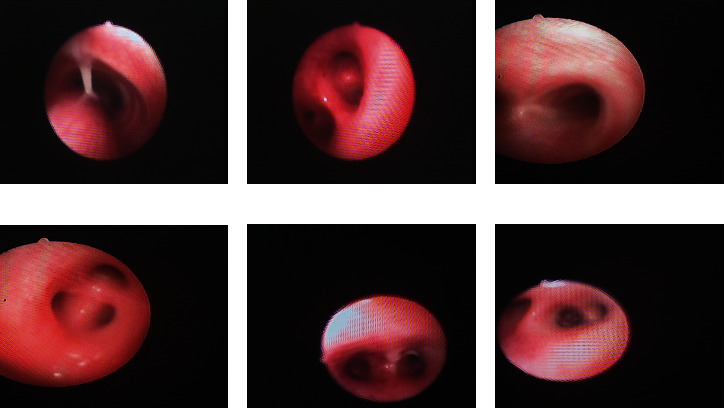
Appearance during fiberoptic bronchoscopy. (a) Thick phlegm in the trachea; (b) inflammation of the tracheal mucosa; (c) pale tracheal mucosa; (d) tracheal mucosa edema; (e) intratracheal phlegm deposit; (f) after bronchoalveolar lavage.

All children with PBB were subject to FOB, except for one child who did not undergo FOB and BAL. The FOB results indicated that for the airway mucosa of children with PBB, the secretions in the trachea, bronchial wall, or lumen increased significantly, with white viscous secretions or suppurative secretions attached to the wall or blocking the lumen (Figure 1(a)). A few may show cellulose-like changes, resulting in lumen clogging that is difficult to remove. The symptoms performance of the children can be divided into two categories: acute and chronic suppurative changes. The acute changes included the main manifestations: mucosal congestion, edema (Figures 1(b) and 1(d)), and increased secretions that may be attached to the wall in a membrane form and may be accompanied by a mucous plug that blocked the lumen (Figure 1(e)). The chronic suppurative changes included pale mucosa and edema (Figure 1(c)). After FOB lavage, most of the secretions were removed (Figure 1(f)). Chest radiography results for children with PBB appeared normal, but CT results indicated that patchy inflammatory shadows were present in some of these children.

FOB results indicated that the secretions were frequently observed in the left main bronchus (78.7%), right lower lobe (64.5%), right upper lobe (62.4%), left lower lobe (59.6%), dorsal segment (46.8%), right middle lobe (39%), right main bronchus (30.5%), left upper lobe (17%), and carina (14.2%). The distribution of sputum in children with PBB was significantly different from that in children without PBB (*P* < 0.05). In addition to sputum, the proliferation of lymphoid follicles in the throat wall of children with PBB (33 (23.4%)) was substantively higher than that for children without PBB (19 (9.2%)) (*P* < 0.05) (Table 4).

### 3.4. Posthospital Discharge Therapy

All children with PBB were treated with antibiotics and nebulization: budesonide suspension (2 mL) plus salbutamol solution (1.5 mL) plus normal saline (2 mL). For children with large patchy shadows on chest radiographs, the clinician will also instruct the patient in chest physiotherapy.

### 3.5. Laboratory Results

After admission, the results of mycoplasma antibody and nucleic acid tests showed no statistically significant difference between the groups of children with vs. without PBB. The results of venous blood tests before bronchoscopy indicated that there were no marked differences in white blood cell, C-reactive protein, and hemoglobin levels between the two groups; however, the blood neutrophil count was significantly higher in the PBB-diagnosed children when compared to non-PBB (*P* < 0.05) (Table 5), which may be attributable to airway inflammation.

Of 141 children with PBB, 85 underwent lung function tests after admission. There were 54 cases of abnormal pulmonary ventilation function, 4 cases of mixed lesions, and 5 cases of restricted lesions, among which 1 case was restricted lesion without classification of severity, 4 cases were mildly restricted lesion. 1 case was small airway dysfunction, and 44 cases were obstructive lesion, among which 13 cases were not classified as severe, 12 cases were mild obstructive lesion, and 2 cases were mild to moderate obstructive lesion. There were 9 cases of moderate obstructive disease, 3 cases of moderate and severe obstructive disease, and 5 cases of severe obstructive disease. Among the 54 children with PBB with abnormal lung function, there were 28 cases with cough-variant asthma, 6 cases with bronchial asthma, and 20 cases with PBB alone.

### 3.6. Pathogens

The presence of bacteria or viruses in the BALF samples was analyzed by direct immunofluorescence. Of these samples, 26 cultures were positive for the presence of bacteria or viruses, including 9 cases with *Streptococcus pneumoniae*, 2 cases with *Escherichia coli*, 5 cases with *Staphylococcus aureus*, 2 cases with *Klebsiella acidophilus*, 2 cases with *Pseudomonas aeruginosa*, 5 cases with Morella, and 1 case with oral *Streptococcus*. There were eight respiratory pathogens detected in the BALF, and 7 cases (5.0%) were nucleic acid positive for mycoplasma.

## 4. Discussion

Chronic cough accounts for a high proportion of the diseases seen through outpatient clinics [4]. A chronic cough frequently occurs in Chinese children [17]. In chronic cough, PBB is the most easily overlooked diagnosis [7]. Although previous studies have shown that PBB is a cause of chronic cough in children [18], there are few studies assessing PBB characteristics, and the pathogenesis and inducement of PBB have been less reported. As a result, PBB is often misdiagnosed as other lung diseases due to a lack of awareness of the disease among pediatricians [19]. Our study describes the typical sputum distribution and tracheal wall changes observed by FOB in children with PBB and highlights the importance of a definitive diagnosis of PBB.

PBB is caused by repeated and persistent bacterial infection in the bronchus [12]. Persistent inflammatory stimulation leads to biofilm formation in the respiratory tract, which affects respiratory mucociliary clearance, systemic immune function, and respiratory malformations (e.g. tracheal chondromalacia) [20]. Clinically, PBB is often misdiagnosed as recurrent pneumonia, asthmatic bronchitis, bronchial asthma, or postinfection cough. Chest radiographs usually show no significant inflammatory response, but some children with comorbidities may see changes around the bronchus on chest radiographs [21]. Granted that diagnostic criteria vary by country, there are still deficiencies and room for improvement in the clinical diagnosis of PBB. Therefore, if evidence of PBB infection is found via FOB on the basis of the typical manifestations, such as the specific locations of sputum, PBB can be clinically diagnosed with higher accuracy. Concurring with published reports, our results indicated that FOB appeared to be safe in children [22]. In our study, the average age of children was 3.9 years, and we observed no adverse effects after FOB. After timely treatment, the cough and phlegm on the auscultation of the lungs disappeared rapidly.

Our study is consistent in many respects with published studies on PBB in children. We also discovered that the percentage of children with PBB having lymphoid follicular hyperplasia was far greater than the percentage of children without PBB. Anatomically, the right bronchus is oriented more vertically and it is shorter than the left bronchus, and thus sputum is likely to accumulate in the former [23]. However, using FOB, we found that the most common sites of accumulated thick sputum in children diagnosed with PBB were the left main bronchus, right lower lobe, right upper lobe, left lower lobe, dorsal segment, right middle lobe, right main bronchus, left upper lobe, and carina. The specific distribution of sputum in the trachea and lungs of children with PBB is inconsistent with assumptions based on human anatomy, demonstrating that sputum distribution as determined through FOB examination may aid the diagnosis of PBB. Indeed, without the manifestations observed using FOB, the clinical manifestations of children with PBB are not specific to the disorder.

Consistent with the diagnostic criteria of PBB, positive bacterial cultures of BALF obtained by FOB can be used to diagnose PBB. However, our results indicated that the positive rate of such cultures for bacteria was extremely low: of 140 children who received FOB combined with BAL, only 26 (19%) of the cultures were positive. Undifferentiated *Haemophilus influenzae* and *Streptococcus pneumoniae* are common respiratory pathogens causing PBB [10]. However, the colonies cultured in this study were mostly *Streptococcus pneumoniae* and *Escherichia coli*. This finding may be attributable to the use of antibiotics by these patients before hospitalization.

Marseglia et al. found that milk protein is a risk factor associated with PBB [24]. We found that, in addition to milk protein, dermatophagoides and eczema may also be risk factors associated with PBB. Therefore, when a child with a history of eczema and milk protein allergies and dermatophagoides develops a chronic cough, PBB should be suspected.

Similar to other studies, the clinical symptoms in most of the children were significantly ameliorated after BAL [25]. Our study also showed that children with patchy opacities observed via CT had good absorption of inflammatory lesions after elective follow-up BAL.

Although 2-week antibiotic therapy is effective for PBB, there is no consensus on the course of its use. In our study, children with PBB showed marked improvement after 2 weeks of therapy with amoxicillin and clavulanate potassium. This is consistent with the findings of Marchant et al. [26]. Budesonide suspension combined with salbutamol solution can inhibit inflammation, reduce clinical symptoms, and reduce pulmonary complications [10]. Donnelly et al. found that 13% of patients required longer antibiotics [21]. However, prolonged use of antibiotics can lead to drug resistance, affecting children's health. The British national pediatric cough guidelines (2008) recommended administering oral antibiotics for 4–6 weeks because some children need longer antibiotic treatment [27]. In the present study, the symptoms of most children were relieved after 2 weeks of antibiotic use, which may be related to the use of antibiotics before hospitalization.

In conclusion, dermatophagoides, milk protein allergies, and eczema were associated with an increased risk of PBB. Thus, when children with these associated risk factors have a chronic cough, PBB should be suspected. The distribution of sputum in the trachea and lungs of children with PBB was specific to PBB. Thus, in addition to the assessment of typical clinical features, diagnosis may be improved by the use of FOB. Early completion of FOB and BAL can improve the clinical symptoms of PBB in children.

## Figures and Tables

**Table 1:** Diagnostic criteria for PBB.

Diagnostic criteria	
PBB-clinical	(1) Chronic wet cough >4 weeks' duration
	(2) Except for other diseases that cause wet cough
	(3) After two weeks of antibiotic treatment, the symptoms improved

PBB-micro	(1) Chronic wet cough lasting for 4 weeks
	(2) BAL fluid culture positive for a respiratory pathogen (bacterial growth ≥10^4^ CFU/mL) obtained during FOB
	(3) After two weeks of oral antibiotics (amoxicillin-clavulanate), the cough was relieved

PBB-extended	Both of the above requiring 4 weeks of antibiotic treatment for cough resolution
Recurrent PBB	More than 3 attacks per year

**Table 2:** Demographic and clinical characteristics of children with vs. without protracted bacterial bronchitis (PBB).

Characteristic	With PBB (*n* = 141), no. (%)	Without PBB (*n* = 206), no. (%)	*P* values
Age (years), median, IQR	3.90 (1.75, 6.03)	3.34 (1.23, 6.00)	−0.969 (0.332)
Weight (kg), median, IQR	17.00 (12.00, 22.00)	15.00 (10.88, 22.00)	−1.349 (0.177)
Male sex	90 (63.8%)	138 (67.0%)	0.371 (0.542)
Cough length (months), median, IQR	2.00 (1.00, 6.00)	2.00 (1.00, 4.00)	−2.317 (0.020)
Feeding method			2.449 (0.294)
Breast	84 (61.8%)	144 (69.9%)	
Formula	34 (25.0%)	41 (19.9%)	
Mixed	18 (13.2%)	21 (10.2%)	
Birth order (firstborn)	96 (68.1%)	127 (63.5%)	0.768 (0.381)
Standard antibiotics used	51 (36.2%)	68 (33.3%)	0.297 (0.586)
Allergy	60 (42.6%)	69 (33.5%)	2.914 (0.086)
Dermatophagoides	25 (17.7%)	14 (6.8%)	10.032 (0.002)
Pollen	3 (2.1%)	3 (1.5%)	(0.690)^a^
Milk protein	27 (19.1%)	16 (7.8%)	9.989 (0.002)
Seafood	5 (3.5%)	2 (1.0%)	(0.125)^a^
Eczema	35 (24.8%)	31 (15.0%)	5.192 (0.023)

^a^Fisher's exact probability method was used. *P* < 0.05 was statistically significant.

**Table 3:** Risk factors associated with protracted bacterial bronchitis.

	Odds ratio (95% confidence interval)	*P* values
Dermatophagoides	2.624 (1.283, 5.369)	0.008
Milk protein	2.452 (1.243, 4.836)	0.010
Eczema	1.763 (1.011, 3.075)	0.046
Length of cough	1.004 (0.979, 1.029)	0.779

**Table 4:** Comparison of bronchoscopy results for children with vs. without protracted bacterial bronchitis (PBB).

	PBB (*n* = 141)	No PBB (*n* = 206)	*P* values
Bronchoscopic sputum	95 (67.4%)	13 (6.3%)	145.607 (<0.001)
Lymphoid follicular hyperplasia	33 (23.4%)	19 (9.2%)	13.213 (<0.001)
Carina	20 (14.2%)	6 (2.9%)	15.343 (<0.001)
Left main bronchus	111 (78.7%)	36 (17.5%)	128.602 (<0.001)
Left upper lobe	24 (17.0%)	16 (7.8%)	7.029 (0.008)
Left lower lobe	84 (59.6%)	62 (30.1%)	29.843 (<0.001)
Right main bronchus	43 (30.5%)	9 (4.4%)	44.853 (<0.001)
Right upper lobe	88 (62.4%)	39 (18.9%)	68.15 (<0.001)
Right middle lobe	55 (39.0%)	27 (13.1%)	31.115 (<0.001)
Right lower lobe	91 (64.5%)	58 (28.2%)	45.225 (<0.001)
Dorsal segment	66 (46.8%)	45 (21.8%)	23.978 (<0.001)

**Table 5:** Laboratory test results for children with vs. without protracted bacterial bronchitis (PBB).

	PBB (*n* = 141)	No PBB (*n* = 206)	*P* values (*P*)
WBC count	7.34 (6.08, 9.40)	7.58 (6.21, 10.34)	−0.848 (0.396)
HgB	124.00 (114.00, 131.00)	121.50 (114.00, 130.00)	−1.366 (0.172)
CRP	0.53 (0.06, 3.75)	0.70 (0.20, 5.94)	−1.497 (0.134)
PCT	0.05 (0.01, 3.70)	0.12 (0.02, 4.30)	−2.564 (0.010)
MP-IgM	29 (20.6%)	38 (18.4%)	0.242(0.623)
MP-DNA	60 (92.3%)	30 (81.1%)	(0.114)a
N%	46.78 ± 15.94	41.30 ± 17.47	−2.908 (0.004)
L%	42.80 ± 15.19	49.38 ± 16.58	3.668 (<0.001)

WBC, white blood cell; HgB, hemoglobin; CRP, C-reactive protein; PCT, procalcitonin; MP-IgM, *Mycoplasma pneumoniae*-specific immunoglobulin M; MP-DNA, *Mycoplasma pneumoniae* DNA; N, neutrophil; L, lymphocyte.

## Data Availability

The data used to support the findings of this study are included within the article.

## Abbreviations

PBB: Protracted bacterial bronchitis
IQR: Interquartile range
FOB: Fiberoptic bronchoscopy
BAL: Bronchoalveolar lavage
BALF: Bronchoalveolar lavage fluid.
